# Hydrogen Peroxide Is Involved in Methane-Alleviated Cadmium Toxicity in Alfalfa (*Medicago sativa* L.) Seedlings by Enhancing Cadmium Chelation onto Root Cell Walls

**DOI:** 10.3390/plants13182639

**Published:** 2024-09-21

**Authors:** Yingying Zhao, Jie Yang, Feiyan Jiang, Gan Zhao

**Affiliations:** 1 College of Life and Health Sciences, Anhui Science and Technology University, Chuzhou 233100, China; zhaoyy@ahstu.edu.cn (Y.Z.); 17764358377@163.com (F.J.); 2 College of Life Sciences, Nanjing Agricultural University, Nanjing 210095, China; 10321117@stu.njau.edu.cn

**Keywords:** NADPH oxidase, subcellular distribution, pectin, Cd translocation factor, xylem sap, low oxygen

## Abstract

Although previous studies have demonstrated that methane (CH_4_) can mitigate the toxicity of cadmium (Cd) in alfalfa seedlings, the CH_4_-rich water used in these studies may create hypoxic conditions, potentially influencing the experimental outcomes. Therefore, this study aimed to investigate whether CH_4_ can reduce Cd toxicity in alfalfa seedlings without the interference of hypoxia and to analyze its underlying mechanisms. Here, it was observed that supplementing oxygen with saturated CH_4_-rich water can significantly alleviate the inhibition of 75 μM CdCl_2_ on the growth of alfalfa (*Medicago sativa* L.) seedlings. Less Cd accumulation was also observed in both root and shoot parts, which could be explained by the CH_4_-altered cell wall components in alfalfa seedling roots, including covalent and ionic soluble pectin, and the degree of demethylation in pectin, thus enabling a higher proportion of Cd binding to the cell walls and reducing the entry of Cd into the cells. The above actions of CH_4_ were accompanied by an increase in hydrogen peroxide (H_2_O_2_) content and NADPH oxidase activity, which could be blocked by the addition of the NADPH oxidase inhibitor diphenylene iodonium (DPI). Taken together, these results implied that exogenously applied CH_4_ could alleviate Cd toxicity in alfalfa seedlings by enhancing Cd chelation onto the root cell walls, which might be closely associated with NADPH oxidase-dependent H_2_O_2_ signals. These findings could provide insight into the mechanism through which CH_4_ alleviates Cd toxicity in alfalfa plants.

## 1. Introduction

Methane (CH_4_), as a candidate gasotransmitter [1], has been found to possess numerous biological functions [2,3]. For example, in animals, CH_4_-rich saline has been shown to ameliorate ischemia/reperfusion injury in the kidney [4], myocardium [5], and liver [6] through pathways involving anti-oxidation, anti-inflammation, and anti-apoptosis. In some plant species, CH_4_-rich water has been shown to promote lateral/adventitious root organogenesis [7,8] and enhance tolerance against various abiotic stresses, including salinity [9], drought stress [10], and cadmium (Cd) toxicity [11,12]. Cd as a non-essential element is highly toxic to nearly all living organisms. It is easily absorbed by plants and transferred to the food chain, thereby threatening the health of not only animals but humans [13,14]. In addition, excessive accumulation of Cd can seriously hinder plant growth, leading to reduced crop yield [15]. In recent years, it was discovered that CH_4_-rich water can alleviate the toxicity of Cd toxicity in alfalfa (*Medicago sativa* L.) seedlings [11,12]. This effect is primarily achieved through the restoration of redox homeostasis and the reduction of Cd absorption pathways.

Previous studies have found that cell walls play a crucial role in enhancing plants’ resistance to Cd toxicity by preventing Cd from entering the root cells [16,17]. Plant cell wall components, including cellulose, hemicellulose, lignin, and pectin, can chelate Cd through their active groups, such as carboxyl, hydroxyl, and thiol groups, thereby reducing the transportation of Cd into the cells [17]. Pectin is the primary component of the cell wall that binds with heavy metals [18,19]. It is widely accepted that pectin is synthesized in the Golgi and then secreted into cell walls in a highly methyl-esterified form. The demethylation of pectin is regulated by pectin methyl esterase (PME) to release carboxyl groups to bind more Cd onto the cell wall [20,21]. After Cd enters the cells, it can be partially loaded into the xylem and transported from the root to the shoot. Heavy metal ATPase 2 (HMA2) has been reported to be involved in the loading of Cd into xylem vessels [22]. Excessive accumulation of Cd in plants can stimulate the overproduction of reactive oxygen species (ROS), leading to oxidative damage in plant cells [11,12]. While ROS can indeed cause oxidative damage to plants, they also serve as ubiquitous signaling molecules that mediate quick responses to stress factors [23,24]. These bursts of ROS are mainly affected by the plasma membrane NADPH oxidases (respiratory burst oxidase homologs (RBOHs)). Because hydrogen peroxide (H_2_O_2_) is relatively stable and can permeate the cell membranes via aquaporins, it was considered to be the main signal of ROS [25]. It has been reported that H_2_O_2_ induced by Cd stress contributes to Cd binding on the cell wall by stimulating pectin biosynthesis and demethylation in rice plants [26].

Although it has been proven that CH_4_ can mitigate Cd toxicity in alfalfa seedlings [11,12], the CH_4_-rich water utilized in these experiments may induce hypoxic conditions, potentially affecting the results. Therefore, the objective of this study was to examine whether Cd toxicity in alfalfa seedlings is alleviated in the absence of hypoxia and to analyze its underlying mechanisms. In this study, it was found that supplementing oxygen with saturated CH_4_-rich water can significantly alleviate the inhibition of Cd on the growth of alfalfa (*Medicago sativa* L.) seedlings. CH_4_ could regulate the distribution of Cd in the cell wall, soluble fraction, and organelle of the root cells while reducing Cd accumulation in alfalfa, making more Cd accumulate on the cell wall. Additionally, these processes were accompanied by a significant increase in H_2_O_2_ content, presenting a new finding. Therefore, we speculated that CH_4_ could alleviate Cd toxicity in alfalfa seedlings, which is potentially linked to H_2_O_2_ signaling and the distribution of CH_4_-regulated Cd in root cell components. By using pharmacological methods combined with laser confocal scanning microscope technology, this study preliminary proved that CH_4_ could alleviate the toxicity of Cd in alfalfa seedlings by promoting the sequestration of Cd in the root cell wall, which requires the participation of H_2_O_2_ signaling. This research may describe a novel regulatory network underlying the molecular mechanisms of CH_4_-alleviated Cd toxicity in alfalfa plants.

## 2. Results

### 2.1. Contribution of CH_4_ to Cd Stress

**Figure 1:**
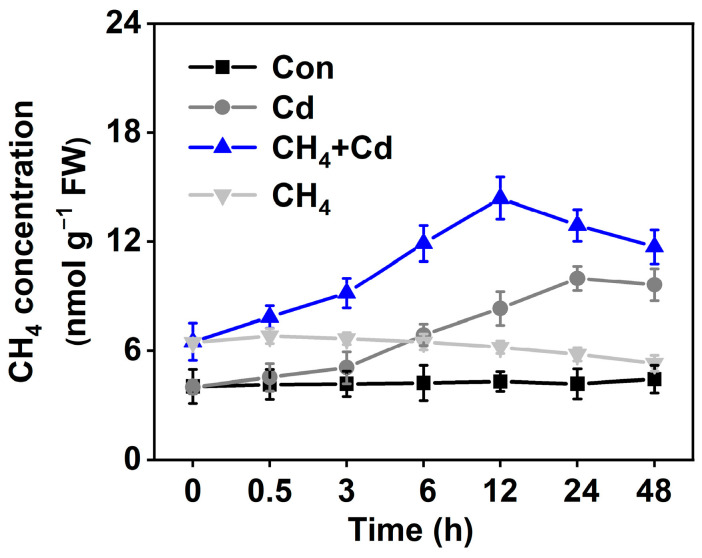
Changes in methane content. After treatment for the indicated time, the methane content of alfalfa (*Medicago sativa* L.) seedlings was detected.

**Figure 2:**
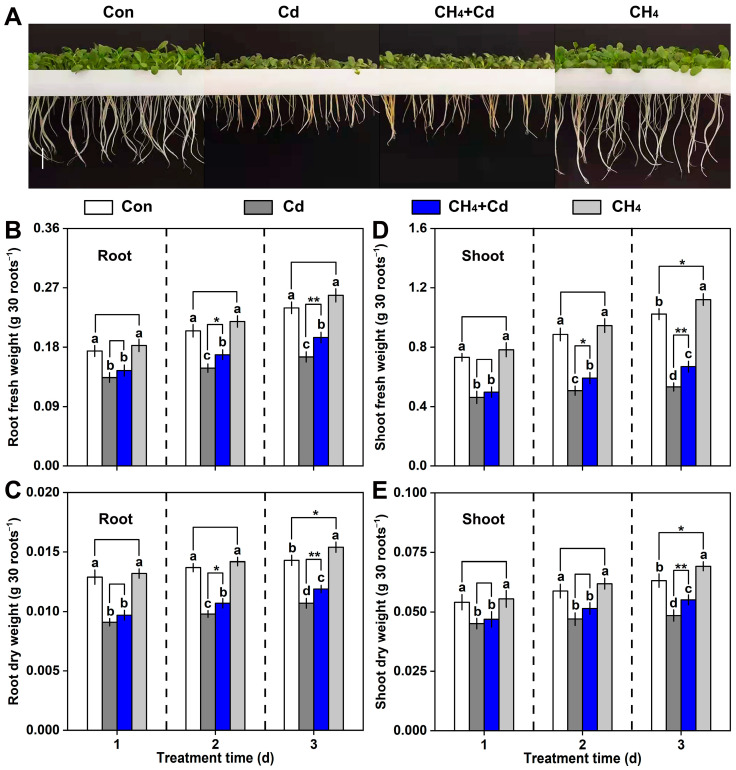
CH_4_-conferred Cd tolerance in alfalfa. Four-day-old seedling pretreated with CH_4_ for 6 h and then transferred to 75 μM condition for another 3 d. (**A**) Phenotypic images after treatment for 3 d. (**B**) Root fresh weight. (**C**) Root dry weight. (**D**) Shoot fresh weight. (**E**) Shoot dry weight. Stress-free groups were used as the control (Con), and bars with different letters denote significant difference at *p* < 0.05 according to Duncan’s multiple range test. Asterisks represent significant difference according to Student’s *t*-test (* *p* < 0.05 and ** *p* < 0.01).

To verify whether CH_4_ confers tolerance against Cd stress in alfalfa (*Medicago sativa* L.) seedlings, CH_4_-rich water supplemented with oxygen was adopted to mimic the potential function of CH_4_. As shown in Figure 1, in the Cd-free condition, the CH_4_ content of CH_4_-pretreated alfalfa seedlings was higher than that of the control alfalfa. Cd stress-elicited CH_4_ production was further observed, which was intensified in the CH_4_-pretreated alfalfa seedlings. As shown in Figure 2, the reduction in fresh and dry weights of roots and shoots caused by Cd stress was partly improved in the CH_4_-pretreated alfalfa seedlings. Under normal conditions, the fresh and dry weights of the roots and shoots were also promoted by CH_4_.

### 2.2. CH_4_-Modulated Cd Accumulation and Subcellular Distribution

**Figure 3:**
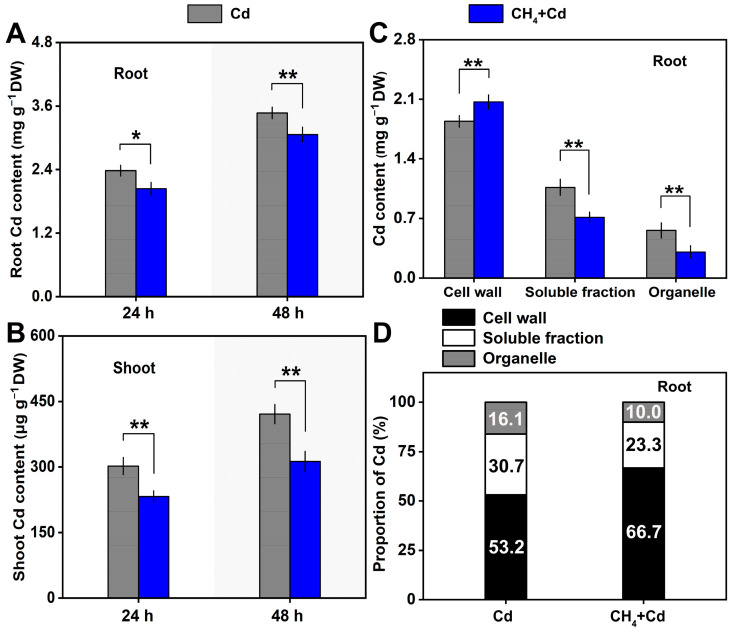
CH_4_-modulated Cd accumulation and subcellular distribution. (**A**) Cd content in roots. (**B**) Cd content in shoots. (**C**) Cd content in the cell wall, soluble fraction, and organelle of alfalfa seedling roots. (**D**) Cd proportion in the cell walls, soluble fractions, and organelles in alfalfa seedling roots. Asterisks represent significant difference according to Student’s *t*-test (* *p* < 0.05 and ** *p* < 0.01).

The Cd accumulation in parts of the roots and shoots of alfalfa was investigated by using an Inductively Coupled Plasma Optical Emission Spectrometer (ICP-OES). As shown in Figure 3A,B, compared to the Cd-treated samples, the Cd content in the root and shoot parts was significantly lower in the CH_4_-pretreated seedlings. The cell walls, soluble fractions, and organellar Cd contents in the roots were also analyzed. However, the results showed that the Cd content in the cell walls of CH_4_-pretreated roots was higher than that in samples treated with Cd alone, while the levels in the soluble and organellar fractions were lower (Figure 3C). The Cd contents in the cell walls and soluble and organellar fractions were 53.2%, 30.7%, and 16.1%, respectively, in the Cd-stressed roots (Figure 3D), but they changed to 66.7%, 23.3%, and 10.0%, respectively, in the CH_4_-pretreated roots.

### 2.3. Cell Wall Modified by CH_4_ under Cd Stress

**Figure 4:**
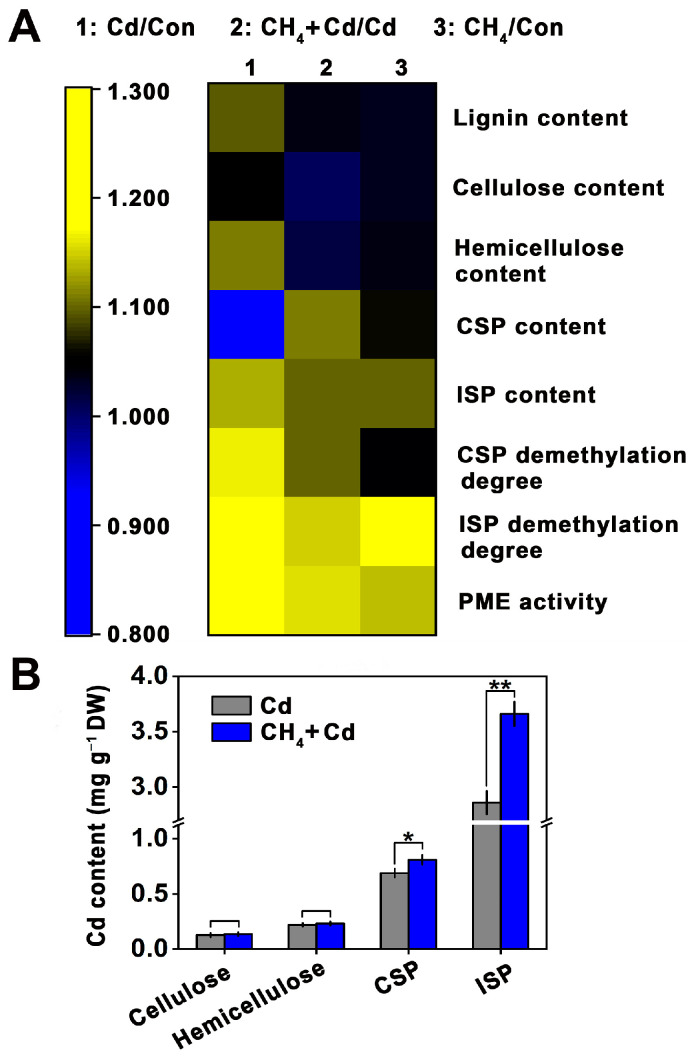
CH_4_-modified cell wall and -altered Cd content in different cell wall components. (**A**) Related content of cell wall compositions, demethylation of CSP and ISP, and PME activities in alfalfa seedling roots (48 h). (**B**) Cd content in root cell wall compositions. Stress-free groups were used as the control (Con). Values are shown as the means ± standard error (SE). Asterisks represent significant difference according to Student’s *t*-test (* *p* < 0.05 and ** *p* < 0.01).

As shown in Figure 4A and Table S1, significantly higher levels of lignin, hemicellulose, and ionic soluble pectin (ISP) were found in alfalfa seedling roots under Cd-stress conditions, along with a lower level of covalent soluble pectin (CSP) in the absence of CH_4_. Subsequent results showed that apart from the increased ISP content, there were no significant changes in the levels of the other components mentioned above in the CH_4_-pretreated alfalfa under Cd-free conditions. Compared to Cd-stressed alfalfa plants, the contents of cellulose and hemicellulose were not changed, but those of CSP and ISP were significantly increased in the CH_4_-pretreated alfalfa seedling roots.

Additionally, the demethylation level and PME activity of pectin in the root cell walls were also determined. As expected, the demethylation and PME activity of pectin were stimulated by Cd and further strengthened in the CH_4_-pretreated alfalfa roots. Under Cd-free conditions, the demethylation level and PME activity of pectin were also promoted in the roots of the CH_4_-pretreated alfalfa.

The Cd contents bound in cellulose, hemicellulose, CSP, and ISP of the root were analyzed (Figure 4B). Compared to the Cd-stressed alfalfa, the Cd contents of CSP and ISP were significantly increased in the CH_4_-pretreated alfalfa. Meanwhile, no significant differences in the Cd-bound binding in cellulose and hemicellulose were observed.

### 2.4. The Function of H_2_O_2_ Signaling in CH_4_-Conferred Cd Stress Tolerance

**Figure 5:**
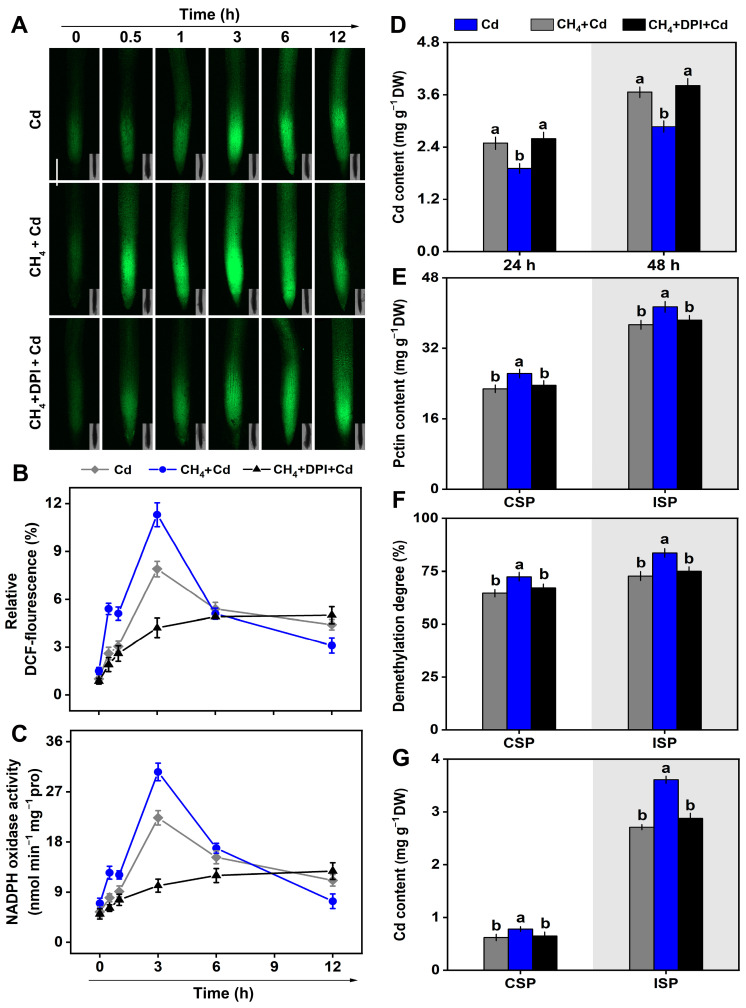
CH_4_-modified cell wall requires NADPH oxidase-dependent H_2_O_2_. (**A**) DCF-dependent H_2_O_2_ fluorescence pictures. (**B**) Related fluorescence densities. (**C**) NADPH oxidase activities in alfalfa roots. (**D**) Cd content in alfalfa root tissues. (**E**) CSP and ISP content in alfalfa seedling roots (48 h). (**F**) Demethylation of CSP and ISP in alfalfa seedling roots (48 h). (**G**) Cd content in CSP and ISP in alfalfa seedling roots (48 h). Values are shown as the means ± standard error (SE), and bars with different letters denote significant difference at *p* < 0.05 according to Duncan’s multiple range test.

Because NADPH oxidase-dependent H_2_O_2_ has been reported to be involved in plant tolerance of Cd stress, H_2_O_2_ generation was analyzed. As shown in Figure 5A–C, the dichlorofluorescein (DCF)-dependent H_2_O_2_ fluorescence and the activities of NADPH oxidase were rapidly increased in the CH_4_-pretreated alfalfa during the 6 h treatment with Cd, peaking at 3 h. After the simultaneous addition of diphenylene iodonium (DPI), an NADPH oxidase inhibitor, the modulated, DCF-dependent H_2_O_2_ fluorescence and NADPH oxidase activities in CH_4_-pretreated alfalfa roots were impaired. The changes in Cd accumulation (Figure 5D) displayed a similar tendency. Subsequently, the relationship between the H_2_O_2_ signal and pectin modulated by CH_4_ was analyzed. As shown in Figure 5E–G, upon Cd stress, CH_4_-intensified CSP and ISP content in the cell wall, the pectin demethylation degree, and Cd content in pectin were totally or partially attenuated after the addition of DPI.

### 2.5. CH_4_-Modulated Cd Translocation from Root to Shoot

**Figure 6:**
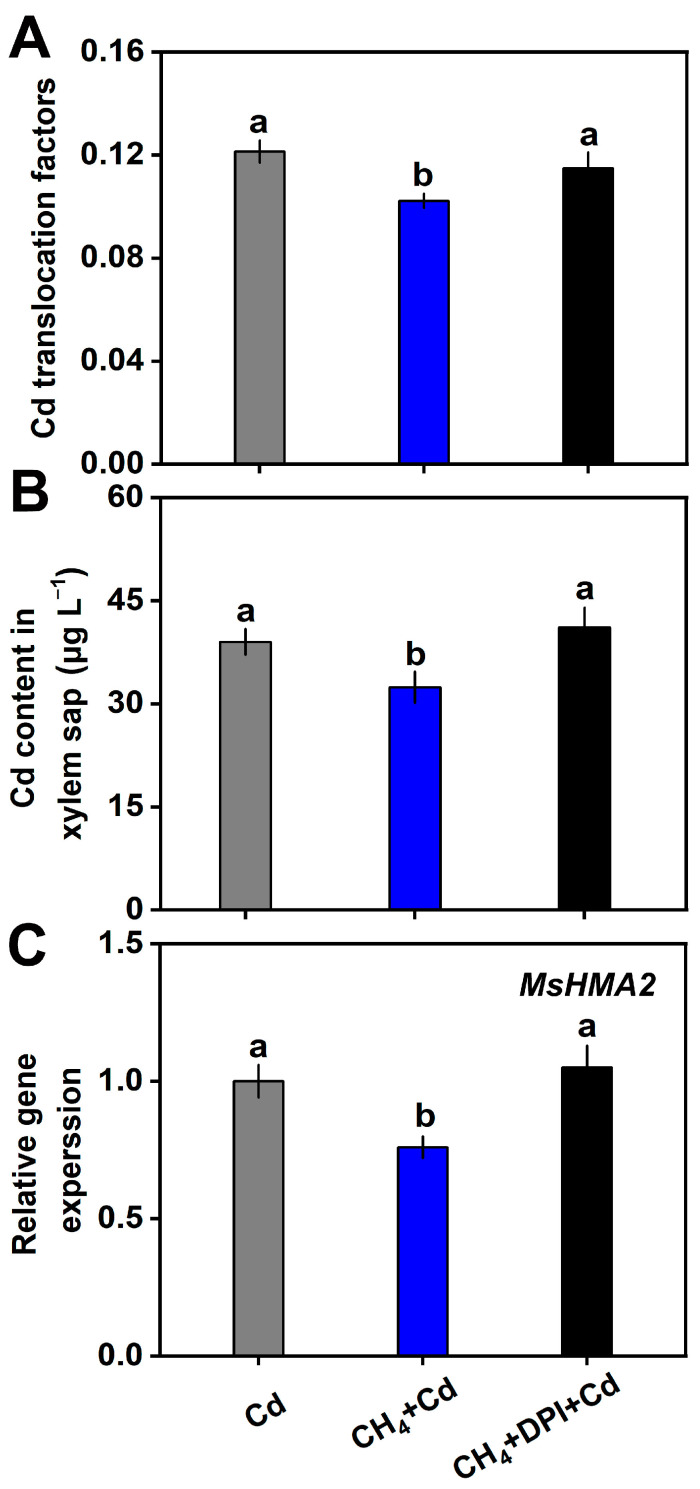
CH_4_-inhibited Cd translocation from roots to shoots requires H_2_O_2_. (**A**) Cd translocation factors (48 h). (**B**) Cd content in xylem sap (48 h). (**C**) The transcript levels of *MsHMA2* in roots (12 h). Values are shown as the means ± standard error (SE), and bars with different letters denote significant difference at *p* < 0.05 according to Duncan’s multiple range test.

The translocation of Cd from the roots to the shoot tissues was further analyzed. As shown in Figure 6A, upon CdCl_2_ stress, the translocation of Cd in alfalfa plants was decreased by CH_4_. The transcripts of *HMA2* in the roots and Cd content in xylem sap were also determined. The results further showed that both *HMA2* expression and Cd content in xylem sap were decreased by CH_4_ and further reversed by the addition of DPI (Figure 6B,C).

## 3. Discussion

Many biological roles of CH_4_ have been discovered [1,2,3], and its non-enzymatic synthesis pathway has been reported [27]. However, the enzymatic synthesis pathway of CH_4_ had not yet been identified. As a result, in current research on the biological roles of CH_4_, the primary method of supplying CH_4_ was by introducing it into solution. Recent studies in the field of plants discovered that saturated CH_4_-rich water can help reduce the toxicity of Cd in alfalfa seedlings. The main mechanism is closely linked to maintaining redox homeostasis and decreasing the accumulation of Cd. However, introducing CH_4_ into a solution might inevitably create a low-oxygen environment, which could affect the experimental results. For example, previous research has shown that low-oxygen conditions slightly alleviate the toxic effects of Cd in alfalfa seedlings. Therefore, to eliminate the interference of low-oxygen conditions in experimental results, our study replenished oxygen to the traditionally prepared CH_4_-rich water. The experimental results showed that without the interference of low oxygen, CH_4_ could alleviate the toxicity of Cd in alfalfa seedlings, manifested as CH_4_-alleviated inhibition of alfalfa seedling growth under Cd stress (Figure 2) and CH_4_-reduced accumulation of Cd in seedlings (Figure 3A,B). This is consistent with previous studies using traditional CH_4_-rich water [11,12]. These findings further revealed that CH_4_, rather than low oxygen, can alleviate the toxicity of Cd in alfalfa seedlings.

Methane can reduce the accumulation of Cd in the roots of alfalfa seedlings while altering the distribution of Cd in the cellular components of the roots, specifically increasing the content of Cd in the cell walls and reducing its content in organelles and soluble proteins (Figure 3C,D). This further reduces the damage caused by Cd to the root cells of alfalfa seedlings, a finding that has not been reported previously. This phenomenon might be attributed to the higher pectin content in the root cell wall and the increased level of pectin demethylation (Figure 4A). Consequently, more Cd is adsorbed onto the cell wall upon entering the cell, which results in reduced levels of Cd in soluble compounds and organelles. Additionally, the decrease in the Cd content in organelles and soluble components might also be associated with the expression of genes encoding metal ion transporters regulated by CH_4_ [11]. The specific mechanisms involved warrant further investigation. It was noteworthy that the mechanism of Cd-toxicity alleviation in alfalfa caused by CH_4_, as found in this study, aligns with the mechanism of Cd-toxicity alleviation in rapeseed caused by boron, as indicated in previous research [28]. This indicates that there might be a relationship between CH_4_ and boron in alleviating Cd toxicity.

During the process of mitigating Cd toxicity through CH_4_, there was an initial increase in H_2_O_2_ content, followed by a subsequent decrease in H_2_O_2_ levels (Figure 5A,B). While previous studies have reported a reduction in H_2_O_2_ content in the roots of alfalfa seedlings under Cd stress induced by CH_4_ [11], the initial increase in H_2_O_2_ levels due to CH_4_ exposure represents a novel discovery. Combining previous research that reported that H_2_O_2_ signaling can promote pectin biosynthesis and demethylation and the release of relative functional groups involved in Cd binding on cell wall pectin, which is beneficial for Cd retention in rice roots [26], this study speculated that H_2_O_2_ signaling might be involved in the CH_4_-mediated mitigation of Cd toxicity. To test this speculation, pharmacological methods combined with fluorescence probe technology were used in this study. The results indicate that the inhibition of alfalfa seedling growth, the reduction of Cd accumulation, and the increase in Cd accumulation in the cell walls mediated by the regulation of pectin through CH_4_ mitigation of Cd stress could be reversed by DPI (Figure 5D–G), a specific inhibitor of NADPH oxidase [29]. NADPH oxidase was identified as the primary source of H_2_O_2_ in this research (Figure 5A–C). These findings offer initial theoretical backing for the hypothesis put forth in this study. The results described above are different from the observation that CH_4_ could diminish late oxidative damage via the inhibition of ROS production in both animals [4,5,6] and plants [9,10,11,12] when supplemented with oxidative stress, thus reflecting the complexity of CH_4_ signaling. Certainly, the involvement of other nonmembrane sources of H_2_O_2_ in our research model cannot be easily ruled out.

It has been reported that H_2_O_2_ can reduce the transfer of Cd from the root to the shoot in rice [30]. By calculating the Cd transfer rate, this study also found that CH_4_ can reduce the transportation of Cd from alfalfa roots to aboveground parts. This phenomenon might be associated with the CH_4_-regulated expression of *HMA2* (Figure 6C) and the distribution of Cd within the root cell components (Figure 3C,D). In this study, by using DPI, we found that the H_2_O_2_ signal was involved in CH_4_’s inhibition of the transfer of Cd from root to shoot.

Collectively, this study preliminarily revealed that CH_4_ can alleviate Cd toxicity in alfalfa seedlings by enhancing the chelation of Cd in the root cell wall, potentially involving H_2_O_2_ signaling. This study was based on pharmacological experiments, and genetic evidence needs to be provided in future research.

## 4. Materials and Methods

### 4.1. CH_4_ Supply and the Determination of CH_4_ Content

CH_4_ was supplied according to a method described previously [11,12], with minor modifications. Briefly, the CH_4_ gas (99.9%, *v*/*v*, Bengbu Lianhua Industrial Gas Co., Ltd., Bengbu, China) was bubbled into quarter-strength Hoagland solutions until the concentration of CH_4_ no longer increased, resulting in a saturated methane-rich water solution. Subsequently, O_2_ (99.99%, *v*/*v*, Bengbu Lianhua Industrial Gas Co., Ltd.) was bubbled into the solution until the original O_2_ concentration was restored. This solution was used for the supply of CH_4_ in the experiment. The O_2_ concentration was monitored by using a dissolved oxygen electrode (DO530, Lohand Biological, Hangzhou, China), as described previously [31].

According to the previously described method [32], the CH_4_ content in seedlings was determined by using a chromatographic system (GC Agilent 7820, Agilent Technologies, Santa Clara, CA, USA) equipped with a Poropak column (1/8 in., 8 foot) and a flame-ionization detector (FID).

### 4.2. Plant Materials, Growth Conditions, and Experimental Design

The growth conditions of alfalfa (*Medicago sativa* L. cv. Victoria) were consistent with the method described previously [11,12]. According to the previous method [11], uniform four-day-old alfalfa seedlings were selected and pretreated with CH_4_ and CH_4_ plus 30 μM of DPI (an inhibitor of NADPH oxidase) [29] for 6 h and then transferred to quarter-strength Hoagland nutrient solution containing 75 μM of CdCl_2_ for the indicated time.

After treatment, the fresh and dry weights of the roots and shoots of alfalfa (per 30 seedlings) were determined. Eighteen roots per treatment were used to check the DCF-dependent H_2_O_2_ fluorescence. A specified fresh or dry weight of each sample was used to measure the contents of Cd, lignin, cellulose, hemicellulose, CSP, and ISP as well as CSP and ISP demethylation degrees, PME activities, and the transcriptional levels. Each experiment was repeated at least three times.

### 4.3. Subcellular Components’ Isolation and Cell Wall Fraction Determination

Subcellular components were separated according to the method described previously [28]. Fresh samples (0.5 g) were thoroughly ground in 8 mL of pre-cooled homogenate (0.25 mM sucrose, 50 mM Tris-HCl buffer, 1 mM disulfide, pH 7.5) and centrifuged at 2000× *g* for 10 min at 4 °C. The precipitate obtained was considered to be the cell wall. The supernatant was then centrifuged at 12,000× *g* for 45 min at 4 °C. The pellet obtained was considered to be the organelle fraction, while the supernatant contained the soluble fraction, including macromolecular organic matter and inorganic ions from the cytoplasm and the vacuole.

After the cell walls were extracted, the lignin content was analyzed by using the modified acetyl bromide method [33]. Additionally, the extraction and determination of cellulose, hemicellulose, covalently bound pectin (CSP), ion-bound pectin (ISP), and PME activity were measured using the corresponding kits from Suzhou Comin Biotechnology Co., Ltd., (Suzhou, China), which were adopted and described in previous studies [26]. The degree of demethylation of CSP and ISP was determined according to the method described previously [34].

### 4.4. Cd Elements’ Detection and Cd Translocation Factors’ Calculation

To remove the Cd adsorbed on the surface of the root system, the root samples were rinsed in 20 mmol L^−1^ of EDTA-Na_2_ solution for 20 min and subsequently washed three times with deionized water [35]. After the samples were oven-dried at 75 °C until a constant weight was achieved and digested using a Digital Block Sample Digestion System (LabTech ED54 DigiBlock, LabTech, Beijing, China), the Cd content in the samples was determined using an ICP-OES (Perkin Elmer Optima 2100, DV; PerkinElmer, Shelton, CT, USA).

Root to shoot Cd translocation factors were calculated according to the following formula, described previously [36]. Cd Translocation Factors = $\frac{Cd\;content\;in\;shoots}{Cd\;content\;in\;roots}$.

### 4.5. Xylem Sap Collection

Xylem sap was collected according to the previous method [37,38], with some modifications. Four-day-old alfalfa plants were pretreated with DPI for 6 h and then transferred to CdCl_2_ conditions for another two days. After cutting the seedlings with a sterilized razor blade approximately 0.2 cm above the roots, the aboveground parts were collected and placed in a 10 mL test tube. Subsequently, the samples were subjected to negative pressure in a vacuum pump for 12 h to collect xylem sap. The Cd element content in xylem sap was analyzed after filtering through 0.45 μm membranes.

### 4.6. Determination of H_2_O_2_ Production and NADPH Oxidase Activity

The fluorescent probe 2′,7′-dichlorofluoresceindiacetate (H_2_DCF-DA) was used to stain H_2_O_2_ in root tissues [35], which were then observed using a Zeiss LSM 800 confocal microscope (Carl Zeiss, Oberkochen, Germany; excitation 488 nm, emission 490–530 nm). The relative H_2_O_2_ dye fluorescence was expressed as values relative to control samples.

NADPH oxidase activity was analyzed according to a previous method [36]. Briefly, 2 g of root samples was used for crude membrane extraction. After adding 15–20 μg of membrane proteins to the assay mixture, the reduction of O_2_^−^ by sodium 3′-[1-[phenylaminocarbonyl]-3,4-tetrazolium]-bis (4-methoxy-6-nitro) ben-zene-sulfonic acid hydrate (XTT) was determined at 470 nm. The NADPH-dependent O_2_^−^ generation rate was calculated using an extinction coefficient of 2.16 × 10^4^ M^−1^ cm^−1^. The amount of protein was determined through the method of Bradford [39].

### 4.7. Quantitative Real-Time PCR (qPCR) Analysis

The qPCR was performed after extracting the total RNA from root tissues to synthesize cDNA. The related gene-specific primers are listed in Supplementary Table S2. The relative transcript levels were calculated by using the 2^−ΔΔCT^ method [40] and presented as values relative to corresponding control samples. The gene transcript levels were normalized to *MSC27* and *Actin2*.

### 4.8. Statistical Analysis

All values were shown as the means ± standard error (SE). The statistical analysis was performed through one-way analysis of variance (ANOVA) followed by Duncan’s multiple range test (*p* < 0.05) or the two-tailed unpaired Student’s *t*-test (*p* < 0.01 and *p* < 0.05) using SPSS 17.0 software.

## 5. Conclusions

In conclusion, this research found that CH_4_ can alleviate Cd toxicity in alfalfa seedlings by promoting Cd chelation onto root cell walls. This mechanism might be mediated by H_2_O_2_ signaling. These findings present a new regulatory network in the molecular mechanism of CH_4_-alleviated Cd toxicity in alfalfa plants.

## Data Availability

The datasets generated and/or analyzed during this study are available from the corresponding author upon request.

## Supplementary Materials

The following supporting information can be downloaded at https://www.mdpi.com/article/10.3390/plants13182639/s1, Table S1: Root cell wall components in alfalfa (*Medicago sativa* L.). Table S2: Primer sequences for qPCR in alfalfa.
