# Supplementary material for: Hydrogen Peroxide Is Involved in Methane-Alleviated Cadmium Toxicity in Alfalfa (Medicago sativa L.) Seedlings by Enhancing Cadmium Chelation onto Root Cell Walls
Source: Plants (Basel). 2024 Sep 21;13(18):2639. doi: 10.3390/plants13182639 (PMC11435170; doi:10.3390/plants13182639)
Supplement: Supplementary file 1 [file plants-13-02639-s001.zip › plants-3159759-supplementary.pdf]

Supplementary Materials

Table S1: Root cell wall components in alfalfa (*Medicago sativa* L.).

	Con→Con	Con→Cd	CH_4→Cd	CH_4→Con
Lignin content (mg g^{-1} DW)	40.59±1.28b	44.49±1.36a	47.09±1.11a	41.96±1.03b
Cellulose content (mg g^{-1} DW)	189.6±8.7a	199.3±8.9a	200.2±9.9a	195.8±7.9a
Hemicellulose content (mg g^{-1} DW)	288.9±9.4b	320.7±7.6a	325.8±10.3a	295.3±8.7b
CSP content (mg g^{-1} DW)	26.15±0.67a	23.32±0.86b	25.85±0.73a	27.68±0.91a
ISP content (mg g^{-1} DW)	32.79±0.73c	37.17±0.62b	40.78±0.85a	36.12±0.81b
CSP demethylation degree (%)	58.68±1.82c	68.34±1.64b	75.09±2.55a	61.25±2.17c
ISP demethylation degree (%)	61.12±1.56c	71.93±1.93b	82.84±2.06a	73.66±1.61b
PME activity (U g^{-1} FW)	0.4912±0.0313c	0.5796±0.0210b	0.6703±0.0389a	0.5591±0.0302b

Table S2: Primer sequences for qPCR in alfalfa.

Primer name	Sequence (5’→3’)
MSC27	F: AGAATGGAATGTTGTGGGAGG
	R: GTCATCAACACCCTCATCTTCTC
Actin2	F: AAAAGGATGCCATGTTGGTG
	R: TAAGTGGAGCCTCAGTTAGAAGTA
MsHMA2	F: TTGTGCCACAGAAGCAACAC
	R: TTCGAGCAGGAACAATCACAGA
